# Age-Related Effects of Olive Oil Polyphenol Ingestion on Oxidation of Low-Density Lipoprotein in Healthy Japanese Men: A Randomized Controlled Double-Blind Crossover Trial

**DOI:** 10.3390/nu16193342

**Published:** 2024-10-01

**Authors:** Shogo Tsujino, Shohei Sadamitsu, Naohisa Nosaka, Tatsuya Fushimi, Yoshimi Kishimoto, Kazuo Kondo

**Affiliations:** 1 Strategic Invention R & D, The Nisshin OilliO Group, Ltd., Yokohama 235-8558, Japan; s-sadamitsu@nisshin-oillio.com (S.S.); n-nosaka@nisshin-oillio.com (N.N.); t-fushimi@nisshin-oillio.com (T.F.); 2 Department of Food Science and Human Nutrition, Setsunan University, Osaka 573-0101, Japan; yoshimi.kishimoto@setsunan.ac.jp; 3 Ochanomizu University, Tokyo 112-8610, Japan

**Keywords:** olive oil polyphenols, malondialdehyde-modified low-density lipoprotein (MDA-LDL), low-density lipoprotein oxidation, extra virgin olive oil, Japanese

## Abstract

Background: The function of olive oil polyphenols in suppressing the oxidation of low-density lipoprotein (LDL) is well-known in Europeans. However, it remains unclear whether olive oil polyphenols exert antioxidant effects in Japanese people. Objectives: The objective of this study was to determine whether the ingestion of olive oil polyphenols suppresses LDL oxidation in the Japanese population and whether this effect depends on age. Methods: This randomized controlled double-blind crossover trial with a 2-week washout enrolled 80 healthy Japanese men aged 35–64 years. Participants ingested either 14 g of extra virgin olive oil containing 5.0 mg of olive oil polyphenols (test food) or 14 g of refined olive oil containing 0.3 mg of olive oil polyphenols (control food) for 3 weeks. The primary outcome was oxidized LDL (malondialdehyde-modified LDL; MDA-LDL). Subgroup analyses based on age (35–50 and 51–64 years) were also performed. Results: In all of the participants (35–64 years), there were no significant differences in MDA-LDL between the control and test groups. However, in the 35–50 years subgroup, ingestion of olive oil polyphenols led to a significantly larger reduction in MDA-LDL as compared with the control group (*p* < 0.025). Conclusions: The significantly lower dietary total polyphenol intake of the 35–50 years subgroup compared to the 51–64 years subgroup suggests that the suppressive function of olive oil polyphenol intake on LDL oxidation in Japanese men is influenced by dietary habits and is more clearly demonstrated in the younger age population with a relatively low total polyphenol intake.

## 1. Introduction

Arteriosclerotic disorders, including ischemic heart disease and peripheral arterial disease, are among the leading causes of death in countries worldwide [1], including Japan [2], and prevention of arteriosclerosis, where the arteries become thick and rigid, is important for extending both life expectancy and healthy life expectancy. The Mediterranean diet is known to be effective in preventing atherosclerosis [3,4,5], and because olive oil is an essential and major vegetable oil in this diet, many studies have investigated the health effects of its consumption [6,7]. In particular, these studies have reported that olive oil consumption decreases the risk of cardiovascular disease [8,9,10] and suppresses the oxidation of low-density lipoprotein (LDL) [11], a key factor in the progression of atherosclerosis.

Specifically, polyphenols present in olive oil are considered to reduce the risk of atherosclerosis because their antioxidant properties have anti-inflammatory effects [12,13]. Representative olive oil polyphenols include tyrosol, hydroxytyrosol, and their derivatives, which are digested and converted to tyrosol and hydroxytyrosol in the gastrointestinal tract [14,15,16]. The polyphenol content decreases during the refining process; therefore, extra virgin olive oil, which is obtained from the fruit by physical processes such as pressing and filtration, has a high polyphenol content, while refined olive oil has a much lower polyphenol content [16,17,18]. Several intervention studies conducted using olive oils with different polyphenol content have found that ingestion of olive oil with higher polyphenol content better suppresses LDL oxidation relative to olive oil with lower content [17,18,19]. For example, Covas et al. conducted a large intervention study in Europe involving 200 healthy men with an average age in their 30s. Oxidized LDL level was evaluated after 3 weeks of ingestion of each of the three olive oils with different olive oil polyphenol content. The result reported that oxidized LDL decreased linearly with increasing olive oil polyphenol content [17].

In Japan, there has been a sharp rise in the import and consumption of olive oil in the past 20 years, fueled by the diversification of food culture and the growing health consciousness of the Japanese people; this increase in consumption of extra virgin olive oil is likely to particularly significant because of expectations of its health benefits [20,21]. To date, however, clinical studies exploring the health benefits of olive oil polyphenols have been conducted mainly in European participants; as far as we know, there have been no similar trials in Japanese individuals.

Comparing the Japanese and European populations, polyphenol intake is almost the same in both groups [22,23,24], but sources differ, being mostly coffee and green tea in Japanese [24,25,26,27], and coffee, fruit (including olives), and red wine in Europeans [28,29]. In addition, it has been reported that daily lipid intake in European populations is higher (Greece: about 110 g; Spain: about 100 g; Italy: about 80 g; Germany: about 100 g; Netherlands: about 100 g; UK: about 95 g (male data)) [30] than in the Japanese (about 70 g (male data)) [31]. Therefore, it is unclear whether olive oil polyphenols exert their antioxidant properties on LDL in Japanese individuals, whose living environments and dietary habits differ from those of Europeans. Furthermore, although aging has been reported to be a major risk factor for atherosclerotic diseases [32], there are few data on whether the effects of olive oil polyphenols differ with age.

The objectives of this study were, therefore, to determine the effect of consuming extra virgin olive oil, which is rich in polyphenols, on LDL oxidation in Japanese individuals and also to determine whether there are differences in effects among individuals of different ages.

## 2. Materials and Methods

### 2.1. Study Design and Ethical Considerations

The present randomized controlled double-blind crossover trial was conducted among healthy adult men in Tokyo, Japan, between 30 January and 21 April 2023. It was conducted under the supervision of a physician by a contract research organization (HUMA R&D Co., Ltd., Tokyo, Japan) at the Takeo Clinic (Tokyo, Japan) in accordance with the Declaration of Helsinki, the Ethical Guidelines for Medical and Biological Research Involving Human Subjects [33], and the Act on the Protection of Personal Information [34]. This study was reviewed and approved by the ethics committees of Yoga Allergy Clinic (approval number: RD11007TK04, approval date: 14 October 2022), and the details were registered with UMIN-CTR before this study commenced (UMIN-ID: UMIN000049473) [35].

### 2.2. Participants

Adult men were recruited from a subject enrollment bank and screened by interview, vital signs (systolic blood pressure, diastolic blood pressure, pulse rate), physical measurements (height, weight, body mass index), blood chemistry (triglyceride (TG), total cholesterol (TC), HDL cholesterol (HDL-C), LDL cholesterol (LDL-C), and malondialdehyde-modified LDL (MDA-LDL)). Women were excluded from this study because estrogen is known to suppress LDL oxidation [36]. Participants who met the selection criteria and had none of the exclusion criteria listed below, were able to comply with the administrative requirements during the study period, and were judged by the study physician to be fit for participation were enrolled in this study.

#### 2.2.1. Selection Criteria

(1) Japanese male aged between 35 and 64 years at the date of consent; (2) a body mass index of 21–30; (3) an LDL-C level of 100–140 mg/dL; (4) non-smoker; (5) an alcohol intake of less than 30 g/day.

#### 2.2.2. Exclusion Criteria

(1) Those receiving medical treatment for a serious disease. (2) Those undergoing exercise or diet therapy under medical supervision. (3) Those allergic to any of the ingredients (wheat, milk protein, soybean, chicken, pork) in the regulated meal. (4) Those with a current or past history of drug or alcohol dependence. (5) Those with current or past mental disorders (e.g., depression) or sleep disorders (e.g., insomnia or sleep apnea). (6) Those working irregular hours due to night shifts. (7) Those performing physical labor for 10 h or more per week. (8) Those with extremely irregular lifestyles (including eating or sleeping). (9) Those who were extremely fussy eaters. (10) Those exercising for physical fitness at least twice a week for at least 30 min. (11) Those with a weight fluctuation of more than ±5 kg within 2 months. (12) Those with serious current or past illnesses (e.g., brain diseases, malignant tumors, immunological diseases, diabetes, liver diseases (hepatitis), renal diseases, cardiac diseases, thyroid diseases, adrenal diseases, and other metabolic diseases). (13) Those who are currently consuming olive oil, health foods, supplements, or medicines that affect antioxidant capacity (e.g., vitamin E and C, coenzyme Q10, beta-carotene, lycopene, anthocyanins, catechins, astaxanthin). (14) Those who had participated in clinical trials within the 3 months prior to the date of consent or had plans to participate in other trials during the study period. (15) Those who had donated more than 200 mL of blood within 1 month or 400 mL of blood within 3 months prior to the date of consent. (16) Those who had difficulty in completing the questionnaires.

### 2.3. Sample Size

Based on the findings of a European clinical trial comparing oxidized LDL levels after 3 weeks of continuous ingestion of a control food or olive oil containing either 7.0 mg (tyrosol equivalent) or 3.1 mg of olive oil polyphenols per day [17], the number of participants required to observe differences was estimated to be more than 29 or 74, respectively. However, given that (1) the present study aimed to investigate 5.0 mg (tyrosol equivalent) per day, (2) the aforementioned study included participants with LDL-C levels of 140 mg/dL or higher (which might have made it easier to observe the effects of olive oil), and (3) there was limited knowledge on the potential influence of Japanese race and dietary habits, it was determined that a larger number of participants should be enrolled. Therefore, the sample size was set at 80.

### 2.4. Test Food and Control Food

The test food was extra virgin olive oil (The Nisshin OilliO Group, Ltd., Tokyo, Japan), which contained 5.0 mg of polyphenols (tyrosol, hydroxytyrosol, and derivatives) per 14 g. The control food was refined olive oil (The Nisshin OilliO Group, Ltd.), which contained 0.3 mg of polyphenols. Olive oil polyphenol content was analyzed according to the method of the International Olive Council [37].

### 2.5. Dietary Survey

Participants completed a daily dietary survey using a Brief-type Self-administered Diet History Questionnaire (BDHQ) [38]. The daily polyphenol intake was calculated by multiplying the food consumption data from each subject’s completed BDHQ with the total polyphenol content of the foods using the Japanese database; the main values from the database are described elsewhere [25,39].

### 2.6. Study Design

Individuals who met the inclusion criteria at screening were enrolled in this study. A third party not involved in the research project (i.e., the individual who allocated the test foods in the clinic) randomly assigned the participants to one of two sequences: in Sequence I, participants ingested the test food first; in Sequence II, they ingested the control food first. After assignment, the same person confirmed that there were no significant differences in MDA-LDL, LDL-C, or age between the two sequence groups. Moreover, they kept the allocation list strictly confidential until the study had finished; thus, blinding was maintained for all parties except the person who allocated the test foods.

**Figure 1:**
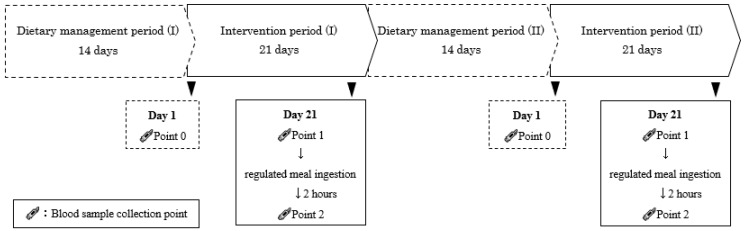
Study protocol. In this crossover study, there was a 14-day dietary management period before intervention periods I and II. During each intervention period, participants ingested the test food every day and recorded their eating and living habits on the dietary and lifestyle questionnaires. Blood samples were collected on Day 1 of each intervention period (Point 0; fasting), Day 21 (Point 1; fasting), and 2 h after ingestion of the regulated meal on Day 21 (Point 2).

The study protocol is shown in Figure 1. Referring to a large intervention study conducted in Europe [17], the intervention period was set at 3 weeks and the washout period at 2 weeks. From 14 days before the first intervention period (I) until the end of the second intervention period (II), the participants underwent dietary and lifestyle management. On Day 1 of this study, participants visited the clinic to provide a fasted baseline blood sample (Point 0). The test (Sequence I) or control (Sequence II) food was then ingested at 14 g per day on a raw for 20 days. On Day 21, participants visited the clinic, where they gave a fasted blood sample (Point 1) and then ingested a regulated meal (energy, 266 kcal; carbohydrate, 22.1 g; protein, 2.6 g; fat, 18.5 g) containing either the test or control food at the designated time. After 2 h, another blood sample was collected (Point 2). Participants then continued on dietary management for 14 days before repeating the intervention for the control (Sequence I) or test (Sequence II) food ingestion (Figure 1).

#### 2.6.1. Dietary Management

(1) Participants were asked to refrain from intake of olive oil, health foods, medicines, and supplements that affect antioxidant capacity (vitamins E and C, coenzyme Q10, beta-carotene, lycopene, anthocyanins, catechins, astaxanthin, etc.). If ingestion of a new medicine/supplement was essential, they were asked to record details on the Lifestyle Questionnaire. Participants were also asked to record any habitual ingestion of unrestricted medicines, supplements, health foods, or functional foods and not to change their usual habits. (2) Participants were requested not to alter their normal intake of foods containing high levels of components that may affect antioxidant capacity (e.g., vegetables, fruits/juices, red wine, beer, cocoa, chocolate, coffee, green tea, salmon, salmon roe, black tea, oolong tea, eggs, fish, and shellfish). Any changes in frequency or amount of consumption should be recorded on the Lifestyle Questionnaire.

#### 2.6.2. Lifestyle Management

During the study period, participants were instructed to keep their body weight as unchanged as possible and comply with the following guidelines. (1) They were requested to maintain a normal lifestyle and to limit any excessive exercise, overeating, and heavy drinking that significantly deviated from their daily lives. (2) They were asked to make as few changes as possible to their lifestyle and environment (e.g., meals, alcohol consumption (<30 g/day), exercise, sleep, and work) before this study. (3) Those with exercise habits were asked not to engage in more strenuous exercise or change the quantity or quality of their exercise. Those with a sedentary lifestyle were asked not to start a new exercise program. (4) Participants were asked not to participate in blood donation. (5) Those undergoing a medical or physical examination were requested to record the information on the Lifestyle Questionnaire.

#### 2.6.3. Specific Compliance before Test Day

(1) Participants were asked to refrain from strenuous exercise in the 3 days prior to the test. (2) They could not drink alcohol on the day before the test. (3) Participants were to keep to the same sleep time as much as possible during intervention periods I and II, especially the day before the test. (4) They were told to fast (water only) from 9:00 p.m. on the day before the test until ingestion of the regulated meal. In addition, fatty foods and sweetened beverages were to be avoided in the last meal/drink before fasting. (5) Participants were instructed to refrain from vaccinations and medical examinations in the 7 days before the test day.

### 2.7. Outcomes

The primary outcome was serum MDA-LDL levels. Secondary outcomes were serum LDL-C, small dense LDL (sd-LDL), and potential antioxidants (PAOs). 

### 2.8. Blood Biochemical Analysis

Serum was obtained by centrifugation (3000 rpm, 10 min). TG was analyzed by the enzymatic method (Cholestest TG; SEKISUI MEDICAL Co., Ltd., Tokyo, Japan) using a JCA—BM8000 (JEOL Ltd., Tokyo, Japan). TC was analyzed using the enzymatic method (Cholestest CHO; SEKISUI MEDICAL Co., Ltd.) using a JCA—BM8000. HDL-C was analyzed using the direct method (CholestestN HDL; SEKISUI MEDICAL Co., Ltd.) using a JCA—BM8000. LDL-C was calculated using a variant of the Friedewald formula [40]. MDA-LDL was analyzed by the ELIZA (Oxidized LDL ELIZA; SEKISUI MEDICAL Co., Ltd.) using microplate reader (SpectraMax; Molecular Devices, San Jose, CA, USA). sd-LDL was analyzed using the direct method (sLDL-EX; Denka Co., Ltd., Tokyo, Japan) using JCA—BM8000. PAO was analyzed by the colorimetric method (antioxidant capacity measurement kit; Nikken SEIL Co., Ltd., Shizuoka, Japan) using microplate reader (DS2; Dynex Technologies Inc, Chantilly, VA, USA).

### 2.9. Adverse Events

During the study period, individuals were asked to record any adverse events daily on the Lifestyle Questionnaire, and the study physician investigated whether there were any causal relationships with the test food.

### 2.10. Statistical Analysis

Nutrient and total polyphenol intakes were checked for normality by the Shapiro–Wilk test. If there was no normality, Mann–Whitney’s U test was performed; if normality was found, equal variances were confirmed with the F test. If equal variances were present, Student’s *t*-test was performed; if not, Welch’s *t*-test was performed.

The baseline values before the intervention (Point 0) were checked for normality by the Shapiro–Wilk test. If there was no normality, a Mann–Whitney U test was performed; if normality was found, ANOVA was performed using a linear model with time, diet, and participants as fixed effects, and intervention effect values were tested.

For each outcome measurement, the change from the baseline value (Point 0) was calculated for fasting (Point 1) and 2 h after ingestion of the regulated food (Point 2) on day 21 of the intervention. Intervention effect values between the test and control groups were used for statistical analysis. The Mann–Whitney U test was used to test the intervention effect values between the two groups.

For testing the intervention effect values for each outcome measure obtained from the blood samples, *p*-value < 0.025 was considered statistically significant, and 0.025 < *p*-value < 0.05 was considered to show tendency towards significance by Bonferroni’s correction for multiplicity due to the fact that two group tests were conducted at fasting (Point 1) and 2 h after ingestion of the regulated food (Point 2) on day 21, respectively. For the other endpoints (nutrient and total polyphenol intakes and the baseline values before the intervention), *p*-value < 0.05 was considered statistically significant, and a *p*-value < 0.05 < 0.1 was considered to show tendency towards significance.

The participants were also subdivided into two subgroups based on age (35–50 years and 51–64 years), and the above statistical analysis was repeated for each age group. In addition, comparisons were made between the younger subgroup (35–50 years) and the older subgroup (51–64 years) for nutrient and total polyphenol intake in the manner presented next.

The Shapiro–Wilk test was performed to confirm normality. If there was no normality, the Mann–Whitney U test was performed; if there was normality, the F test was performed to confirm equal variances. If equal variances were found, Student’s *t*-test was performed; if not, Welch’s *t*-test was performed.

The validity of the crossover study design was verified by testing the carryover effect of repetition for the outcome measures where a significant difference was observed.

Basic data analysis was conducted using Microsoft Excel for Office365 MSO version 2308 (Microsoft Japan Corporation, Tokyo, Japan); R statistical software, v4.1.0 for Windows (R Core Team, Vienna, Austria), was used for statistical processing.

## 3. Results

### 3.1. Participants

**Figure 2:**
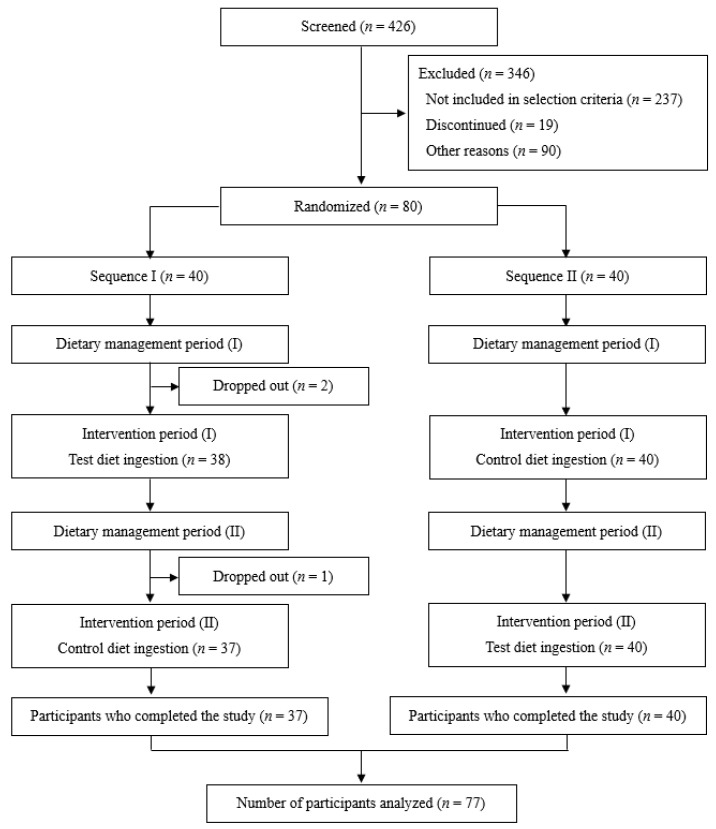
Flowchart of the study. Among 426 candidates screened, 346 were excluded, and 80 were enrolled in the study. Participants were randomly assigned to either Sequence I (ingestion of test food first) or Sequence II (ingestion of control food first). Two participants in Sequence I dropped out during dietary management period I, and one in Sequence I dropped out during dietary management period II; thus, data from 77 participants were analyzed.

Among 426 individuals who gave consent and were screened for eligibility, 80 were enrolled in this study. They were randomly assigned to two sequences, resulting in 40 participants in Sequence I and 40 in Sequence II. Subsequently, two participants in Sequence I dropped out during dietary management period I, and one in Sequence I dropped out during dietary management period II; thus, data from 77 participants were analyzed (Figure 2). The baseline clinical data of the participants are summarized in Table 1.

### 3.2. Adverse Events

No adverse events attributable to the ingestion of the test foods were observed during the study period.

### 3.3. Nutrient Intake

The average daily nutrient and total polyphenol intake is shown in Table 2. There were no significant differences between the control and test groups. 

We divided the population into two groups based on age, with a stratified analysis of the younger subgroup (35–50 years, *n* = 38) and the older subgroup (51–64 years, *n* = 39). Daily nutrient and total polyphenol intakes are shown in Table 3. Total polyphenol intake was significantly higher in the older subgroup (51–64 years) than in the younger subgroup (35–50 years), although there were no significant differences between the test and control groups.

### 3.4. Outcomes

The baseline values before the intervention (Point 0) are shown in Table 4. There was no significant difference in any baseline values, but serum TG showed a tendency towards lower (*p* = 0.07) in the test group.

The values of each outcome for all participants are summarized in Table 5. There were no significant differences between the two groups in any outcomes.

The results of the age-based analysis are summarized in Table 6 for the younger subgroup (35–50 years) and in Table 7 for the older subgroup (51–64 years). Among the younger subgroup, MDA-LDL was significantly more reduced from baseline in the test group as compared with the control group at 2 h after ingestion of the regulated food on day 21 (Point 2). Serum sd-LDL was significantly higher in the test group as compared with the control group at fasting on day 21 (Point 1) and Point 2 among the younger subgroup. 

Serum PAO, which indicates antioxidant capacity in the blood, tended to be higher in the test group at Points 1 and 2 among the younger subgroup. Serum TG was significantly higher in the test group compared to the control group at Point 2. Among older subgroups, there were no significant differences between the test and control groups in any outcomes.

There were no carryover effects for any of the outcome measures for which significant differences were found.

## 4. Discussion

The health functions of olive oil, a major source of lipids in the Mediterranean diet, have long been the focus of research studies [6,7], and many have shown that polyphenols specific to olive oil have various beneficial properties, such as antioxidant and anti-inflammatory effects [41]. In Europe, several projects have been conducted to evaluate the health function of extra virgin olive oil, such as EUROLIVE [17], SOLOS [18], VOHF [42], and NUTRAOLEUM [43], and although each project has different conditions, all of the results support the ability of olive oil polyphenols to inhibit the oxidation of blood lipids. In addition, the European Food Safety Authority (EFSA) supports the health claim that “olive oil polyphenols contribute to the protection of blood lipids from oxidative stress” [44,45]. Thus, the health functions of olive oil polyphenols have been incorporated into European health and nutrition policy.

Although consumption of extra virgin olive oil has been increasing in Japan because of its potential health benefits, to our knowledge, there have been no intervention studies conducted in Japanese populations to investigate the function of olive oil polyphenol intake in inhibiting the oxidation of blood lipids.

**Figure 3:**
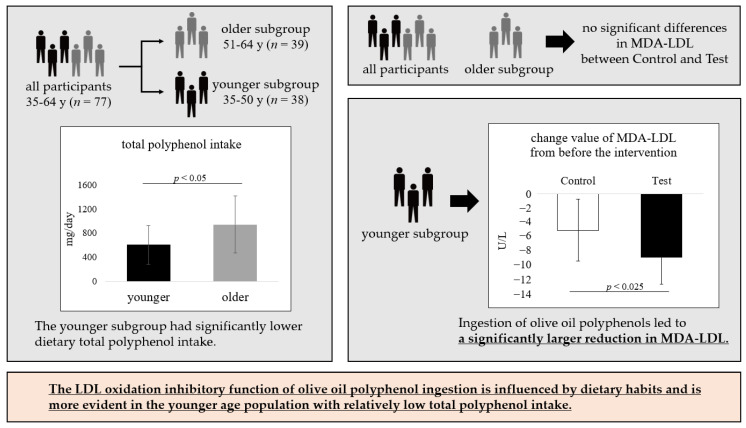
Overview of this study.

In this study, we evaluated the LDL oxidation inhibitory function of olive oil polyphenol ingestion in Japanese men. Previous intervention studies conducted in Europe investigated an olive oil intake of 20 g/day or more [17,18,19]. Given the lower reference intake for fat in the Japanese population [46], we considered that an intervention of 20 g/day would be excessive. Therefore, participants were given 14 g of olive oil per day containing 5.0 mg of olive oil polyphenols. The results showed no significant difference in MDA-LDL for the overall aged 35–64 years. However, in participants with similar pre-intervention MDA-LDL in the test group by age (older subgroup: 104.4 ± 5.8 U/L, younger subgroup: 94.8 ± 4.3 U/L), a significant reduction of MDA-LDL by olive oil polyphenol ingestion was observed in the younger subgroup (35–50 years). Compared to the older subgroup, the younger subgroup had significantly lower dietary total polyphenol intake, suggesting that in Japanese men, the LDL oxidation inhibitory function of olive oil polyphenol intake is influenced by dietary habits and is more evident in the younger age population with relatively low total polyphenol intake (Figure 3).

Aging is a risk factor for atherosclerotic diseases [32]. In fact, the Japan Atherosclerosis Society guidelines for the prevention of atherosclerotic cardiovascular disease state that aging is the strongest risk factor for atherosclerotic diseases [47]. To our knowledge, however, few studies have investigated whether the health benefits of olive oil polyphenols differ depending on age. In this study, the suppression of LDL oxidation by olive oil polyphenol ingestion was significant in the younger subgroup and not in the older subgroup. Given this observation, we further analyzed differences between the test and control foods for age-based subgroups (35–45 years, *n* = 19; 35–55 years, *n* = 54), which showed that MDA-LDL was significantly lowered in the test group as compared with the control group at Point 2 in both subgroups (Supplementary Tables S1 and S2). Furthermore, the effect size was 0.78 for the 35–45-year-old subgroup and 0.43 for the 35–55-year-old subgroup, suggesting that ingestion of olive oil polyphenols clearly led to LDL oxidation suppression in the younger participants. Intake of polyphenols in the Japanese population has been reported to increase with age [48]. In this study, consumption was about 600 mg/day in the younger subgroup and about 950 mg/day in the older subgroup (Table 3), and it was significantly higher in the older subgroup than in the younger subgroup. Because coffee and green tea are the main sources of polyphenols in the Japanese diet [24,25,26,27], we carried out a post hoc analysis comparing coffee, green tea, and black/oolong tea intake between the two subgroups, which showed that intake of coffee and green tea was significantly higher among the older than the younger subgroup (Supplementary Table S3). Thus, the older subgroup had a higher intake of polyphenols, especially those derived from coffee and green tea, in their normal diet, which may have been a confounding factor, making the effect of olive oil polyphenols ingestion less detectable.

The possible mechanisms by which olive oil ingestion suppresses LDL oxidation are (1) the antioxidant capacity of the polyphenols themselves and (2) the enhanced expression of oxidative stress defense genes via NF-E2-related factor 2 (Nrf2). First, the olive oil polyphenols are tyrosol, hydroxytyrosol, and derivatives, which undergo hydrolysis in the stomach and small intestine to tyrosol and hydroxytyrosol [14]. Tyrosol and hydroxytyrosol are absorbed in a dose-dependent manner [49,50,51], with peak blood concentrations occurring about 1 h after their ingestion [52]. Some reports also suggest that ingestion of olive oil increases their absorption [53,54]. Tyrosol and hydroxytyrosol each have antioxidant activity and have been demonstrated to suppress LDL oxidation in vivo and in vitro [55,56]. Furthermore, a single 25 g dose of olive oil rich in olive oil polyphenols was found to significantly reduce blood levels of oxidized LDL in a European study [19]. 

Second, the transcription factor Nrf2 regulates the expression of genes encoding antioxidants such as glutathione-S-transferase and heme oxygenase-1 [57], and olive oil polyphenols are known to modulate Nrf2 transcriptional activity and enhance antioxidant capacity [58]. In this study, the suppression of LDL oxidation by olive oil ingestion was observed only at Point 2, suggesting that single and continuous ingestion of olive oil polyphenols produces beneficial effects in a combined manner.

sd-LDL, a type of LDL with a small particle size and high density, is considered more atherogenic than LDL due to its higher penetration into the vessel wall, lower binding affinity for LDL receptors, longer residence time in the blood, and greater susceptibility to oxidation [59,60,61,62]. In this study, sd-LDL showed little change in values before and after ingestion of the test food, but the value was significantly higher in the test group than in the control group in the younger subgroup. However, sd-LDL and blood TG levels are known to be positively correlated [63,64]; therefore, the difference in sd-LDL level observed between the two food groups might be attributed to TG level. We note, however, that there was a tendency toward a significant difference between the two groups in TG level at baseline (Point 0) and that postprandial blood collection was conducted only at one point 2 h after the meal. Further studies will be needed to evaluate in detail the effect of ingestion of extra virgin olive oil on changes in blood lipids in the future.

Because extra virgin olive oil is not produced by a refining process, it contains not only more polyphenols but also more lipid peroxides (e.g., TG hydroperoxides) as compared with refined oils [48,65]. The peroxide value of the control food was 0.2, whereas that of the test food was 4.4. Although both amounts are small, recent reports suggest that ingestion of small amounts of TG hydroperoxides can cause the oxidation of lipids in vivo [66]. In this study, no adverse events attributable to ingestion of the test foods were recorded, and the intervention of extra virgin olive oil reduced MDA-LDL levels, suggesting that the level of lipid peroxides in extra virgin olive oil does not affect the function of the polyphenols.

This study has some limitations. First, the effect of the interaction of olive oil polyphenols with other dietary components is unknown because the BDHQ was used to conduct the dietary survey. Second, the study participants were healthy men aged 35–64 years, and the effects on individuals 34 years and younger or 65 years and older remain unknown. Third, the intervention period was 3 weeks, representing a short-term dietary intervention. Last, only one postprandial blood sample was collected; therefore, the changes in blood lipids over time were unknown.

Japan has one of the longest life expectancies in the world, but atherosclerotic diseases constitute a major factor that hinders the average life expectancy and healthy life expectancy of the Japanese people. In this study, Japanese men whose dietary habits are low in polyphenol intake have shown that incorporating olive oil into their diets may contribute to the prevention of atherosclerosis. In addition, because olive oil is more often eaten with raw vegetables and fruits than other cooking oils, incorporating it into the diet may contribute to an increase in the intake of dietary fiber and potassium, which are considered low in the Japanese people [46]. From these perspectives, we believe that it is significant for Japanese people to incorporate olive oil into their diets, and we expect that longer-term intervention studies will be conducted in the future.

## 5. Conclusions

The results of this randomized controlled double-blind crossover trial showed that ingestion of extra virgin olive oil containing 5.0 mg of olive oil polyphenols resulted in significantly lower MDA-LDL levels (*p* < 0.025) in the younger subgroup (35–50 years), although no significant difference was observed in the Japanese male population (35–64 years). The significantly lower dietary total polyphenol intake of the younger subgroup compared to the older subgroup (51–64 years) suggests that the suppressive function of olive oil polyphenol intake on LDL oxidation in Japanese men is influenced by dietary habits and is more clearly demonstrated in the younger age population with a relatively low total polyphenol intake.

## Figures and Tables

**Table 1:** Baseline clinical characteristics of the participants (*n* = 77) ^1^.

Characteristics		
Age	years	50.4 ± 7.7
BMI	kg/m^2^	24.1 ± 2.1
TG	mg/dL	92.7 ± 41.8
LDL-C	mg/dL	126.2 ± 9.4
HDL-C	mg/dL	60.0 ± 13.4
MDA-LDL	U/L	113.6 ± 31.9

^1^ Values are given as mean ± standard deviation. BMI, body mass index; TG, triglyceride; LDL-C, LDL cholesterol; HDL-C, HDL cholesterol; MDA-LDL, malondialdehyde-modified LDL.

**Table 2:** Mean daily nutrient intake (*n* = 77) ^1^.

		Control	Test
Energy	kcal	1758 ± 496	1754 ± 498
Carbohydrate	g	218.8 ± 72.5	219.7 ± 72.1
Protein	g	61.5 ± 22.2	61.0 ± 22.3
Fat	g	64.7 ± 20.0	64.0 ± 20.1
Saturated fatty acid	g	14.9 ± 5.7	14.8 ± 5.7
Monounsaturated fatty acid	g	29.4 ± 7.6	29.2 ± 7.7
Polyunsaturated fatty acid	g	14.1 ± 4.8	13.8 ± 4.9
Vitamin E	mg	9.9 ± 2.6	9.8 ± 2.6
β-Carotene	mg	2.1 ± 1.5	2.0 ± 1.5
Total polyphenol	mg	777 ± 461	783 ± 420

^1^ Values are given as mean ± standard deviation. No significant difference between the two groups for any of the nutrients.

**Table 3:** Mean daily nutrient intake for each subgroup ^1^.

		35–50 Years (*n* = 38)		51–64 Years (*n* = 39)	
		Control	Test	Control	Test
Energy	kcal	1647 ± 357	1637 ± 375	1866 ± 587	1869 ± 575
Carbohydrate	g	203.0 ± 51.4	204.9 ± 52.5	234.2 ± 86.3	234.2 ± 85.4
Protein	g	57.7 ± 20.8	57.3 ± 23.3	65.2 ± 23.1	64.5 ± 21.0
Fat	g	61.0 ± 17.3	59.5 ± 18.5	68.3 ± 21.9	68.5 ± 20.8
Saturated fatty acid	g	14.3 ± 5.0	14.2 ± 5.1	15.5 ± 6.4	15.4 ± 6.2
Monounsaturated fatty acid	g	28.0 ± 6.8	27.4 ± 7.4	30.7 ± 8.1	30.9 ± 7.6
Polyunsaturated fatty acid	g	12.9 ± 4.1	12.3 ± 4.2	15.2 ± 5.2	15.3 ± 5.0
Vitamin E	mg	9.1 ± 2.0	8.9 ± 2.0	10.6 ± 2.9	10.7 ± 2.9
β-Carotene	mg	1.8 ± 1.5	1.8 ± 1.6	2.3 ± 1.5	2.2 ± 1.5
Total polyphenol	mg	600 ± 323	619 ± 330	950 ± 511 *	942 ± 441 *

^1^ Values are given as mean ± standard deviation. Significant difference as compared with younger subgroup * (*p* < 0.05).

**Table 4:** Study outcomes for all participants at Point 0 (*n* = 77) ^1^.

Outcome		Control	Test	*p* Value
MDA-LDL	U/L	96.1 ± 3.2	99.7 ± 3.6	0.55
sd-LDL	mg/dL	33.3 ± 1.5	32.9 ± 1.6	0.55
PAO	µmol/L	1278.3 ± 20.1	1273.8 ± 21.7	0.66
TG	mg/dL	101.3 ± 7.1	97.8 ± 5.7 †	0.07
LDL-C	mg/dL	128.0 ± 2.2	127.2 ± 2.2	0.45
HDL-C	mg/dL	59.6 ± 1.5	60.1 ± 1.5	0.29

^1^ Values are given as mean ± standard error. Tendency toward intervention effect as compared with control group † (*p* < 0.1). MDA-LDL, malondialdehyde-modified LDL; sd-LDL, small dense LDL; PAO, potential antioxidant; TG, triglyceride; LDL-C, LDL cholesterol; HDL-C, HDL cholesterol. Point 0: a fasted baseline blood sample collected on Day 1 of each intervention period.

**Table 5:** Change value from Point 0 for all participants (*n* = 77) ^1^.

Outcome			Control	Test	*p* Value
MDA-LDL	U/L	Point 1 Point 2	−2.6 ± 3.0 −2.4 ± 3.1	−7.1 ± 3.2 −6.1 ± 2.9	0.88 0.053
sd-LDL	mg/dL	Point 1 Point 2	−2.4 ± 0.6 −1.8 ± 0.7	−1.0 ± 0.8 −0.2 ± 0.8	0.10 0.10
PAO	µmol/L	Point 1 Point 2	41.3 ± 11.3 26.6 ± 11.8	43.8 ± 13.6 31.9 ± 15.3	0.66 0.30
TG	mg/dL	Point 1 Point 2	−10.6 ± 5.0 15.4 ± 5.0	−1.2 ± 4.5 22.0 ± 4.5	0.37 0.07
LDL-C	mg/dL	Point 1 Point 2	−2.1 ± 1.8 −3.4 ± 1.7	−2.0 ± 1.5 −2.8 ± 1.6	0.46 0.66
HDL-C	mg/dL	Point 1 Point 2	0.1 ± 0.7 −0.2 ± 0.7	0.4 ± 0.6 0.2 ± 0.6	0.37 0.76

^1^ Values are given as mean ± standard error. MDA-LDL, malondialdehyde-modified LDL; sd-LDL, small dense LDL; PAO, potential antioxidant; TG, triglyceride; LDL-C, LDL cholesterol; HDL-C, HDL cholesterol. Point 0: a fasted baseline blood sample collected on Day 1 of each intervention period; Point 1: a fasted blood sample collected on Day 21 of each intervention period; Point 2: a blood sample collected 2 h after ingestion of the regulated meal on Day 21 of each intervention period.

**Table 6:** Change value from Point 0 for participants aged 35–50 years (*n* = 38) ^1^.

Outcome			Control	Test	*p* Value
MDA-LDL	U/L	Point 1 Point 2	−3.7 ± 4.0 −5.0 ± 4.3	−8.6 ± 3.8 −8.8 ± 3.7 *	0.21 0.01
sd-LDL	mg/dL	Point 1 Point 2	−3.2 ± 0.9 −2.7 ± 1.0	1.0 ± 1.1 * 1.5 ± 1.1 *	0.001 0.003
PAO	µmol/L	Point 1 Point 2	35.3 ± 16.4 28.0 ± 17.3	68.5 ± 18.1 † 60.3 ± 20.3 †	0.03 0.03
TG	mg/dL	Point 1 Point 2	−21.7 ± 8.8 3.5 ± 8.2	4.9 ± 4.8 † 29.7 ± 5.3 *	0.03 0.01
LDL-C	mg/dL	Point 1 Point 2	−0.7 ± 2.9 −2.0 ± 2.8	−1.0 ± 2.3 −2.1 ± 2.3	0.40 0.68
HDL-C	mg/dL	Point 1 Point 2	0.8 ± 0.8 0.3 ± 0.8	0.7 ± 0.9 0.3 ± 0.9	1 0.83

^1^ Values are given as mean ± standard error. Significant difference in intervention effect as compared with control group * (*p* < 0.025, Bonferroni correction for multiple comparisons). Tendency toward intervention effect as compared with control group † (*p* < 0.05, Bonferroni correction for multiple comparisons). MDA-LDL, malondialdehyde-modified LDL; sd-LDL, small dense LDL; PAO, potential antioxidant; TG, triglyceride; LDL-C, LDL cholesterol; HDL-C, HDL cholesterol. Point 0: a fasted baseline blood sample collected on Day 1 of each intervention period; Point 1: a fasted blood sample collected on Day 21 of each intervention period; Point 2: a blood sample collected 2 h after ingestion of the regulated meal on Day 21 of each intervention period.

**Table 7:** Change value from Point 0 for participants aged 51–64 years (*n* = 39) ^1^.

Outcome			Control	Test	*p* Value
MDA-LDL	U/L	Point 1 Point 2	−1.6 ± 4.4 0.1 ± 4.5	−5.5 ± 5.3 −3.5 ± 4.4	0.17 0.84
sd-LDL	mg/dL	Point 1 Point 2	−1.7 ± 0.8 −0.9 ± 0.9	−2.9 ± 1.1 −1.9 ± 1.1	0.30 0.54
PAO	µmol/L	Point 1 Point 2	46.8 ± 15.6 25.2 ± 16.4	19.8 ± 19.8 4.3 ± 22.1	0.15 0.54
TG	mg/dL	Point 1 Point 2	0.2 ± 4.2 26.9 ± 5.3	−7.2 ± 7.4 14.4 ± 7.2	0.41 0.84
LDL-C	mg/dL	Point 1 Point 2	−3.4 ± 2.0 −4.8 ± 2.1	−3.0 ± 2.0 −3.4 ± 2.1	0.84 0.84
HDL-C	mg/dL	Point 1 Point 2	−0.6 ± 1.1 −0.6 ± 1.1	0.1 ± 1.0 0.2 ± 1.0	0.21 0.53

^1^ Values are given as mean ± standard error. MDA-LDL, malondialdehyde-modified LDL; sd-LDL, small dense LDL; PAO, potential antioxidant; TG, triglyceride; LDL-C, LDL cholesterol; HDL-C, HDL cholesterol. Point 0: a fasted baseline blood sample collected on Day 1 of each intervention period; Point 1: a fasted blood sample collected on Day 21 of each intervention period; Point 2: a blood sample collected 2 h after ingestion of the regulated meal on Day 21 of each intervention period.

## Data Availability

Data described in the manuscript, code book, and analytic code will be made available upon request.

## Supplementary Materials

The following supporting information can be downloaded at https://www.mdpi.com/article/10.3390/nu16193342/s1. Table S1: Change value from Point 0 for participants aged 35–45 years (*n* = 19); Table S2: Change value from Point 0 for participants aged 35–55 years (*n* = 54); Table S3: Beverage intake; Material S1: Lifestyle Questionnaire.
