# Supplementary material for: Age-Related Effects of Olive Oil Polyphenol Ingestion on Oxidation of Low-Density Lipoprotein in Healthy Japanese Men: A Randomized Controlled Double-Blind Crossover Trial
Source: Nutrients. 2024 Oct 1;16(19):3342. doi: 10.3390/nu16193342 (PMC11478568; doi:10.3390/nu16193342)
Supplement: Supplementary file 1 [file nutrients-16-03342-s001.zip › Supplementary Material.pdf]

Supplementary

Supplementary Material S1. Lifestyle Questionnaire

ID:

Age:

Date:

Q1. Did you ingest a test food today?

Q2. What was your weight today?

Q3. Did you have any health problems today? If yes, please provide "Details of Symptom" and "Cause".

Q4. Did you take (use) any medicines (including eye drops, compresses, or applications) today? If you have taken (used) a medicine, please provide the name of the medicine and the dose.

Q5. Did you exercise today? If yes, please fill in the "Details of exercise" and "Duration (minutes)".

Q6. Did you consume alcohol today? If yes, please indicate the type and volume (ml) of alcohol consumed.

Q7. Did you consume any health foods, supplements, or foods for specified health uses today? If yes, please indicate the name of the product and the amount consumed.

Q8. Did you increase or decrease your intake of foods that affect antioxidant capacity significantly today? Please select "Yes" only if there was a significant increase or decrease, and indicate the name of the product and the amount of increase or decrease.

Q9. Were there any other events that changed your life? If yes, please fill in the "Details".
