# Supplementary material for: Age-Related Effects of Olive Oil Polyphenol Ingestion on Oxidation of Low-Density Lipoprotein in Healthy Japanese Men: A Randomized Controlled Double-Blind Crossover Trial
Source: Nutrients. 2024 Oct 1;16(19):3342. doi: 10.3390/nu16193342 (PMC11478568; doi:10.3390/nu16193342)
Supplement: Supplementary file 1 [file nutrients-16-03342-s001.zip › Supplementary Tables_rev.pdf]

Supplementary

Supplementary Table S1. Change value from Point 0 for participants aged 35–45 years (n=19) [1]

Outcome			Control	Test	p value
MDA-LDL	U/L	Point 1	1.5 ± 5.7	-10.4 ± 5.0	0.38
		Point 2	4.9 ± 6.1	**-16.7 ± 8.2***	0.001
sd-LDL	mg/dL	Point 1	-2.6 ± 1.3	0.9 ± 1.8	0.39
		Point 2	-2.1 ± 1.4	0.6 ± 2.8	0.38
PAO	µmol/L	Point 1	23.6 ± 24.7	**84.9 ± 26.8†**	0.04
		Point 2	40.7 ± 28.8	85.7 ± 57.2	0.14
TG	mg/dL	Point 1	-23.3 ± 10.9	**6.2 ± 5.7†**	0.04
		Point 2	3.0 ± 10.7	**33.0 ± 12.1***	0.02
LDL-C	mg/dL	Point 1	0.2 ± 4.6	**-5.2 ± 3.6†**	0.04
		Point 2	-2.0 ± 4.7	-11.4 ± 7.2	0.14
HDL-C	mg/dL	Point 1	1.4 ± 1.4	1.2 ± 1.4	1
		Point 2	0.7 ± 1.3	-0.2 ± 4.3	1

[1] Values are given as mean ± standard error. Significant difference in intervention effect as compared with control group* ($p < 0.025$, Bonferroni correction for multiple comparisons). Tendency toward intervention effect as compared with control group † ($p < 0.05$, Bonferroni correction for multiple comparisons). MDA-LDL, malondialdehyde-modified LDL; sd-LDL, small dense LDL; PAO, potential antioxidant; TG, triglyceride; LDL-C, LDL cholesterol; HDL-C, HDL cholesterol

Point 0: a fasted baseline blood sample collected on Day 1 of each intervention period; Point 1: a fasted blood sample collected on Day 21 of each intervention period; Point 2: a blood sample collected 2 hours after ingestion of the regulated meal on Day 21 of each intervention period.

Supplementary

Supplementary Table S2. Change value from Point 0 for participants aged 35–55 years (n=54) [1]

Outcome			Control	Test	p value
MDA-LDL	U/L	Point 1	-1.6 ± 3.3	-10.2 ± 4.1	0.29
		Point 2	-0.1 ± 3.7	**-7.7 ± 6.2***	0.001
sd-LDL	mg/dL	Point 1	-2.9 ± 0.8	**-0.1 ± 1.0†**	0.03
		Point 2	-2.4 ± 0.9	**0.2 ± 2.4†**	0.03
PAO	μmol/L	Point 1	35.3 ± 14.8	49.1 ± 16.1	0.16
		Point 2	30.9 ± 15.6	**43.1 ± 33.9†**	0.03
TG	mg/dL	Point 1	-17.6 ± 6.5	**5.8 ± 4.4***	0.01
		Point 2	7.0 ± 6.2	**28.4 ± 9.6***	0.002
LDL-C	mg/dL	Point 1	0.4 ± 2.2	-1.9 ± 1.7	0.08
		Point 2	-1.0 ± 2.2	-3.4 ± 4.1	0.48
HDL-C	mg/dL	Point 1	0.5 ± 0.8	0.0 ± 0.8	0.72
		Point 2	0.0 ± 0.7	-0.6 ± 2.4	0.59

[1] Values are given as mean ± standard error. Significant difference in intervention effect as compared with control group* ($p < 0.025$, Bonferroni correction for multiple comparisons). Tendency toward intervention effect as compared with control group † ($p < 0.05$, Bonferroni correction for multiple comparisons). MDA-LDL, malondialdehyde-modified LDL; sd-LDL, small dense LDL; PAO, potential antioxidant; TG, triglyceride; LDL-C, LDL cholesterol; HDL-C, HDL cholesterol

Point 0: a fasted baseline blood sample collected on Day 1 of each intervention period; Point 1: a fasted blood sample collected on Day 21 of each intervention period; Point 2: a blood sample collected 2 hours after ingestion of the regulated meal on Day 21 of each intervention period.

Supplementary

Supplementary Table S3. Beverage intake [1]

		Overall (n=77)		35–50 years (n=38)		51–64 years (n=39)	
		Control	Test	Control	Test	Control	Test
Coffee	g	215 ± 186	211 ± 175	163 ± 144	165 ± 142	**265 ± 209***	**255 ± 194***
Green tea	g	144 ± 175	155 ± 179	95 ± 130	110 ± 140	**191 ± 200***	**199 ± 203***
Black tea / Oolong tea	g	53 ± 92	53 ± 93	45 ± 82	46 ± 82	60 ± 102	59 ± 102

[1] Values are given as mean ± standard deviation. No significant difference between Control and Test for any of the beverage in each group. Significant difference as compared with younger subgroup* ($p < 0.05$).
